# Draft genome sequence of *Caminibacter mediatlanticus* strain TB-2^T^, an epsilonproteobacterium isolated from a deep-sea hydrothermal vent

**DOI:** 10.4056/sigs.2094859

**Published:** 2011-09-23

**Authors:** Donato Giovannelli, Steven Ferriera, Justin Johnson, Saul Kravitz, Ileana Pérez-Rodríguez, Jessica Ricci, Charles O’Brien, James W. Voordeckers, Elisabetta Bini, Costantino Vetriani

**Affiliations:** 1 Department of Biochemistry and Microbiology, Rutgers University, New Brunswick, NJ, USA; 2 Institute of Marine and Coastal Sciences, Rutgers University, New Brunswick, NJ, USA; 3 Institute for Marine Science - ISMAR, National Research Council of Italy, Ancona, ITALY; 4 J. Craig Venter Institute, 9704 Medical Center Drive, Rockville, Maryland, USA; 5 Institute for Environmental Genomics, Department of Botany and Microbiology, University of Oklahoma, Norman, OK, USA

**Keywords:** *Epsilonproteobacteria*, thermophiles, free-living, anaerobes, chemolithoautotrophy, *Nautiliales*, deep-sea hydrothermal vent

## Abstract

*Caminibacter mediatlanticus* strain TB-2^T^ [1], is a thermophilic, anaerobic, chemolithoautotrophic bacterium, isolated from the walls of an active deep-sea hydrothermal vent chimney on the Mid-Atlantic Ridge and the type strain of the species. *C. mediatlanticus* is a Gram-negative member of the *Epsilonproteobacteria* (order *Nautiliales*) that grows chemolithoautotrophically with H_2_ as the energy source and CO_2_ as the carbon source. Nitrate or sulfur is used as the terminal electron acceptor, with resulting production of ammonium and hydrogen sulfide, respectively. In view of the widespread distribution, importance and physiological characteristics of thermophilic *Epsilonproteobacteria* in deep-sea geothermal environments, it is likely that these organisms provide a relevant contribution to both primary productivity and the biogeochemical cycling of carbon, nitrogen and sulfur at hydrothermal vents. Here we report the main features of the genome of *C. mediatlanticus* strain TB-2^T^.

## Introduction

*Caminibacter mediatlanticus* type strain TB-2^T^ (=DSM 16658^T^=JCM 12641^T^) is an epsilonproteobaterium isolated from the walls of an active deep-sea hydrothermal vent on the Mid-Atlantic Ridge [1]. *C. mediatlanticus* is part of the recently proposed order *Nautiliales* [2], which comprises three genera: *Nautilia*, *Caminibacter* and *Lebetimonas*. All *Nautiliales* cultured are thermophilic chemolithoautotrophs and have been isolated from deep-sea hydrothermal vents. The genus *Caminibacter* includes three described species: *C. hydrogeniphilus*, the type strain for this genus [3], *C. profundus* [2], and *C. mediatlanticus* [1]. All three *Caminibacter* species are thermophilic (55- 60 °C) and conserve energy by coupling the oxidation of hydrogen to the reduction of nitrate and sulfur. *C. profundus* can also grow microaerobically (0.5% O_2_) [2]. The genus *Nautilia* includes four species: *N. lithotrophica* [4], *N. profundicola*, whose genome was recently sequenced [5,6], *N. abyssi* [7] and *N. nitratireducens* [8]. While all *Nautilia* spp. couple hydrogen oxidation to sulfur reduction, *N. nitratireducens* can also use nitrate, thiosulfate and selenate as terminal electron acceptors [8]. The genus *Lebetimonas* includes a single species, *L. acidiphila*, a sulfur-respiring chemolithoautotroph [9]. Here we present a summary of the features of *C. mediatlanticus* strain TB-2^T^ and a description of its genome.

## Classification and features

**Figure 1:**
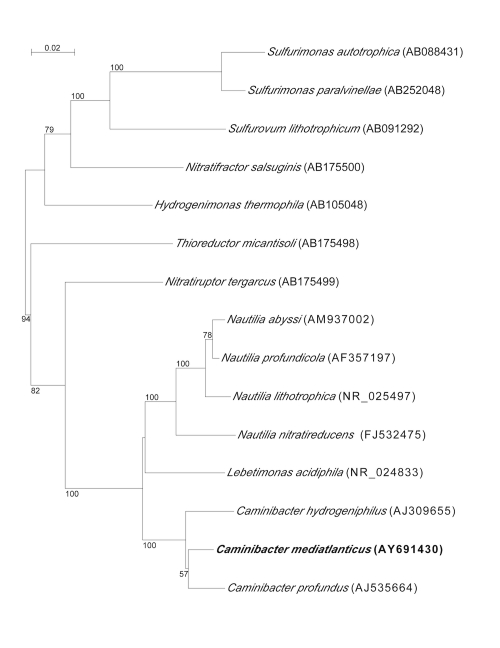
Phylogenetic position of *Caminibacter mediatlanticus* strain TB-2^T^ relative to type strains of *Epsilonproteobacteria* isolated from deep-sea hydrothermal vents. Sequences were aligned automatically using CLUSTAL X and the alignment was manually refined using SEAVIEW [10,11]. Neighbor-joining trees were constructed with Phylo_Win, using the Jukes-Cantor correction [12]. Bootstrap values (>50%) based on 500 replications. Bar, 0.02 substitutions per nucleotide position.

*C. mediatlanticus* strain TB-2^T^ was isolated from the Rainbow vent field on the Mid-Atlantic Ridge (36° 14’ N, 33° 541 W). *Caminibacter* sp. strain TB-1 [1], *C. profundus* and *C. hydrogeniphilus* are the closest relatives to *C. mediatlanticus*, with a 16S rRNA gene similarity of 99%, 96.3% and 95.9%, respectively. The phylogenetic position of *C. mediatlanticus* relative to all the known type strains of *Epsilonproteobacteria* isolated from deep-sea hydrothermal vents is shown in Figure 1.

**Figure 2:**
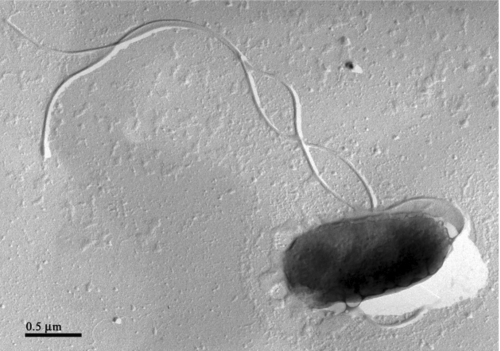
Electron micrograph of a platinum shadowed cell of *Caminibacter mediatlanticus* strain TB-2 ^T^ showing multiple flagella. Bar, 0.5 μm.

The cells of *C. mediatlanticus* are Gram-negative rods of approximately 1.5 x 0.75 µm, motile by mean of one to three polar flagella (Figure 2 and Table 1). On solid media, the cells form small brownish colonies. Growth occurs between 45 and 70˚C, 10 and 40 g NaCl L^-1^ and pH 4.5 and 7.5. Optimal growth conditions are 55˚C, 30 g NaCl l^-1^ and pH 5.5 (generation time 50 min). Growth occurs under strictly anaerobic, chemolithoautotrophic conditions in the presence of H_2_ and CO_2_ with nitrate or sulfur as electron acceptors and the formation of ammonia or hydrogen sulfide, respectively. Oxygen, selenate, arsenate, thiosulfate and sulfite are not used as terminal electron acceptors. No chemoorganoheterotrophic growth has been reported. Evidence that *C. mediatlanticus* fixes CO_2_ via the reductive tricarboxylic acid (rTCA) cycle was obtained by the detection, by PCR, of the gene encoding for the ATP citrate lyase, a key enzyme of the cycle, and by the determination of the specific activities of the rTCA enzymes [19]. The genomic G + C content of *C. mediatlanticus* is 27.13 mol%.

**Table 1:** Classification and general features of *C. mediatlanticus* strain TB-2^T^ according to the MIGS recommendations [13]

**MIGS ID**	**Property**	**Term**	**Evidence code**
	Current classification	Domain *Bacteria*	TAS [14]
		Phylum *Proteobacteria*	TAS [15]
		Class *Epsilonproteobacteria*	TAS [16,17]
		Order *Nautiliales*	TAS [2]
		Family *Nautiliaceae*	TAS [2]
		Genus *Caminibacter*	TAS [3]
		Species *Caminibacter mediatlanticus*	TAS [1]
		Type strain TB-2	TAS [1]
	Gram stain	negative	TAS [1]
	Cell shape	short rod	TAS [1]
	Motility	motile	TAS [1]
	Sporulation	non-sporulating	TAS [1]
	Temperature range	45°C - 70°C	TAS [1]
	Optimum temperature	55 °C	TAS [1]
MIGS-6.3	Salinity	opt.: 30 g NaCl l^-1^ (range 10-40 g NaCl l^-1^)	TAS [1]
MIGS-22	Oxygen	obligate anaerobe	TAS [1]
	Carbon source	CO_2_	TAS [1]
	Energy source	H_2_	TAS [1]
	Terminal electron acceptor	NO_3_, S_0_	TAS [1]
MIGS-6	Habitat	marine, deep-sea hydrothermal vents	TAS [1]
MIGS 14	Pathogenicity	not reported	NAS
	Biosafety level	1	NAS
	Isolation	deep-sea hydrothermal vent, black smoker	TAS [1]
MIGS-15	Biotic relationship	free living	TAS [1]
MIGS-4	Geographic location	Mid-Atlantic Ridge, Rainbow vent field	TAS [1]
MIGS-5	Sample collection time	July 2001	TAS [1]
MIGS-4.1	Latitude	36° 14’ N	TAS [1]
MIGS-4.2	Longitude	33° 54’ W	TAS [1]
MIGS-4.3	Depth	2305 m	TAS [1]
MIGS-4.4	Altitude	not applicable	

Evidence codes - TAS: Traceable Author Statement (i.e., a direct report exists in the literature); NAS: Non-traceable Author Statement (i.e., not directly observed for the living, isolated sample, but based on a generally accepted property for the species, or anecdotal evidence). These evidence codes are from the Gene Ontology project [18]. If the evidence code is IDA, then the property was directly observed, for the purpose of this specific publication, for a live isolate by one of the authors, or an expert or reputable institution mentioned in the acknowledgements.

### Chemotaxonomy

None of the classical chemotaxonomic features (peptidoglycan structure, cell wall sugars, cellular fatty acid profile, respiratory quinones, or polar lipids) are known for *C. mediatlanticus* strain TB-2^T^.

## Genome sequencing information

### Genome project history

The genome of *C. mediatlanticus* strain TB-2^T^ was selected for sequencing in 2005, during phase two of the Microbial Genome Sequencing Project of the Gordon and Betty Moore Foundation, and it was sequenced at the J. Craig Venter Institute. It was the first genome of an *Epsilonproteobacterium* from deep-sea hydrothermal vents to be sequenced. *C. mediatlanticus* was selected because it is a thermophilic member of the *Epsilonproteobacteria*, which, as a group, represent a significant fraction of the chemosynthetic communities inhabiting the deep-sea hydrothermal vents [20, 21]and because of its ability to fix CO_2_ under strictly anaerobic conditions [1]. The draft genome sequence was completed in November 2006 and presented for public access on June 19, 2007. The NCBI accession number is ABCJ00000000.1 and consists of 35 contigs (ABCJ01000001-ABCJ01000035). Table 2 shows the project information and its association with MIGS version 2.0 compliance [22].

**Table 2:** Genome sequencing project information

**MIGS ID**	**Property**	**Term**
MIGS-31	Finishing quality	Draft
MIGS-28	Libraries used	Plasmids and cosmids
MIGS-29	Sequencing platforms	Sanger/pyrosequencing hybrid
MIGS-31.2	Fold coverage	8×
MIGS-30	Assemblers	Celera
MIGS-32	Gene calling method	GeneMark and Glimmer
	Genome Database release	J. Craig Venter Institute
	Genbank ID	ABCJ00000000.1
	Genbank Date of Release	June 19, 2007
	GOLD ID	Gi01407
	Project relevance	Chemosynthetic ecosystems, CO_2_ fixation, Thermophiles

### Growth conditions and DNA isolation

*C. mediatlanticus* was grown in modified SME medium at 55°C under a H_2_/CO_2_ gas phase (80:20; 200 kPa) with CO_2_ as the carbon source and nitrate as the electron acceptor, as described by Voordeckers et al. [1]. Genomic DNA was isolated from 1–1.5 g of pelleted cells using an extraction protocol that involved a phenol:chloroform:isoamyl alcohol(50:49:1) step followed by isopropanol precipitation, as described by Vetriani et al. [23].

### Genome sequencing and assembly

Two genomic libraries with insert sizes of 4 and 40 kbp were constructed from the genomic DNA of *C. mediatlanticus* as described in Goldberg et al. [24]. The resulting plasmid and fosmid clones were sequenced at the J. Craig Venter Institute from both ends to provide paired-end reads and an 8× coverage. The Celera assembler was used to generate contigs and reconstruct the draft genome [25].

### Genome annotation

The genome sequence was analyzed using the Joint Genome Institute IMG system [26], the RAST (Rapid Annotation using Subsystem Technology) server [27], the GenDB annotation program [28] at the Center for Genome Research and Biocomputing at Oregon State University, and the NCBI Prokaryotic Genomes Automatic Annotation Pipeline.

The annotation of the draft genome was done using the Prokaryotic Genomes Automatic Annotation Pipeline of the National Center for Biotechnology Information [29]. The PGAAP combines HMM-based gene prediction methods with a sequence similarity-based approach, and compares the predicted gene products to the non-redundant protein database, Entrez Protein Clusters, the Conserved Domain Database, and the COGs (Clusters of Orthologous Groups).

Gene predictions were obtained using a combination of GeneMark and Glimmer [30-32]. Ribosomal RNAs were predicted by sequence similarity, using BLAST against the non-redundant nucleotide database and/or using Infernal and Rfam models. The tRNAscan-SE [33] was used to find tRNA genes. The predicted CDS were then searched using the NCBI nonredundant protein database. The predicted protein set and major metabolic pathways of TB-2^T^ were searched using the KEGG, SwissProt, COG, Pfam, and InterPro protein databases implemented in the IMG and GenDB systems. Additional gene prediction analysis and manual functional annotation was performed within the IMG and using the Artemis software (release 13.0, Sanger Institute).

## Genome properties

The genome consists of a 1,663,618 bp long circular chromosome with a 27.13 mol% G + C content (Table 3). Of the 1,894 genes predicted, 1,826 were protein-coding genes. Of these, 1,180 were assigned to a putative function, while the remaining genes were annotated as coding for hypothetical proteins. In the genome of *C. mediatlanticus*, 84 protein-coding genes belong to 38 paralogous families, corresponding to a gene content redundancy of 4.44%. The properties and the statistics of the genome are summarized in Table 3. The distribution of genes into Clusters of Orthologous Groups (COGs) functional categories is shown in Table 4.

**Table 3:** Genome statistics

**Attribute**	**Value**	**% of total^a^**
Size (bp)	1,663,618	
G+C content (bp)	451,320	27.13
Coding region (bp)	1,583,997	95.21
Total genes^b^	1,894	
RNA genes	68	3.59
Protein-coding genes	1,826	96.41
Genes in paralog clusters	84	4.44
Genes assigned to COGs	1,461	77.14
Genes assigned in Pfam domain	1,371	72.39
Genes connected to KEGG pathways	630	33.26
Genes with signal peptides	214	11.30
Genes with transmembrane helices	400	21.12
Paralogous groups	38	2.01

a) The total is based on either the size of the genome in base pairs or the total number of protein coding genes in the annotated genome.

b) no pseudogenes found.

**Table 4:** Number of genes associated with the 25 general COG functional categories

**Code**	**Value**	**%age**^a^	**Description**
J	130	7.12	Translation	
K	53	2.9	Transcription	
L	84	4.6	Replication, recombination and repair	
D	21	1.15	Cell cycle control, mitosis and meiosis	
V	14	0.77	Defense mechanisms	
T	79	4.33	Signal transduction mechanisms	
M	108	5.91	Cell wall/membrane biogenesis	
N	58	3.18	Cell motility	
U	52	2.85	Intracellular trafficking and secretion	
O	73	4	Posttranslational modification, protein turnover, chaperones	
C	116	6.35	Energy production and conversion	
G	50	2.74	Carbohydrate transport and metabolism	
E	128	7.01	Amino acid transport and metabolism	
F	52	2.85	Nucleotide transport and metabolism	
H	87	4.76	Coenzyme transport and metabolism	
I	34	1.86	Lipid transport and metabolism	
P	66	3.61	Inorganic ion transport and metabolism	
Q	14	0.77	Secondary metabolites biosynthesis, transport and catabolism	
R	153	8.38	General function prediction only	
S	89	4.87	Function unknown	
-	365	19.99	Not in COGs	

a) The total is based on the total number of protein coding genes in the annotated genome.

### Reconstruction of the rTCA cycle for CO_2_ fixation from the genome sequence of *C. mediatlanticus* strain TB-2^T^

**Figure 3:**
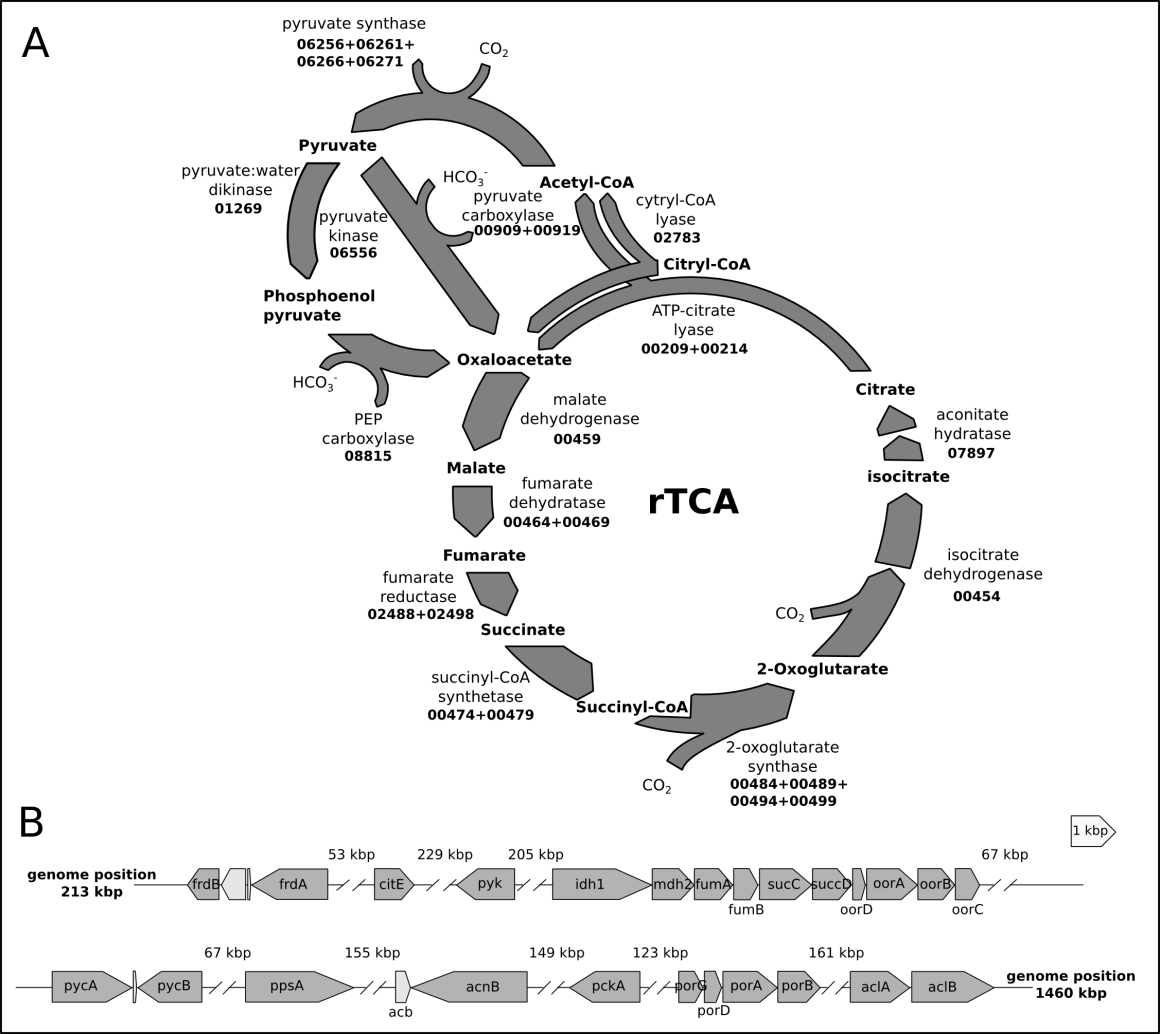
Reconstruction of the rTCA cycle and related gene clusters in *C. mediatlanticus* strain TB-2^T^. A) rTCA cycle. Enzymes are identified by the corresponding gene locus in the *C. mediatlanticus* genome (CMTB2_gene number). B) Structure of the gene clusters encoding for enzymes involved in rTCA cycle. ORF present within the same clusters are shown in light gray. The distance between clusters is reported in thousands of base pairs (kbp). Genes are oriented according to their direction and drawn to scale. *frdBA*: fumarate reductase; *citE*: citril-CoA lyase; *pyk*: pyruvate kinase; *idh1*: monomeric isocitrate dehydrogenase; *mdh2*: malate dehydrogenase; *fumAB*: fumarate hydratase; *sucCD*: succinyl-CoA synthetase; *oorDABC*: 2-oxoglutarate ferredoxin synthase; *pycAB*: pyruvate carboxylase; *ppsA*: pyruvate water dikinase; *acb*: bifunctional aconitate hydratase/methyl isocitrate dehydratase; *acnB*: aconitate hydratase; *pckA*: phosphoenol pyruvate carboxy kinase; *porGDAB*: pyruvate ferredoxinoxido reductase/synthase; *aclBA*: ATP-citrate lyase.

*C. mediatlanticus* strain TB-2^T^ is an obligate anaerobic, hydrogen-dependent chemolithoautotroph. In this bacterium, CO_2_ fixation occurs via the reductive tricarboxylic acid (rTCA) cycle [19]. By fixing CO_2_ in the absence of oxygen, *C. mediatlanticus* is completely independent from photosynthetic processes, and therefore this bacterium is a true primary producer in the deep ocean (in contrast to aerobic chemosynthetic bacteria, which ultimately depend on photosynthesis-derived oxygen for their energy metabolism). In Figure 3 we show a reconstruction of the rTCA cycle and the organization of the rTCA cycle-related genes in the genome of *C. mediatlanticus*.
